# The Impact of the Spectral Radiation Environment on the Maximum Absorption Wavelengths of Human Vision and Other Species

**DOI:** 10.3390/life11121337

**Published:** 2021-12-03

**Authors:** Samuel Konatham, Javier Martín-Torres, Maria-Paz Zorzano

**Affiliations:** 1 Department of Computer Science, Electrical and Space Engineering, Luleå University of Technology, 97187 Luleå, Sweden; javier.martin-torres@abdn.ac.uk (J.M.-T.); zorzanomm@cab.inta-csic.es (M.-P.Z.); 2 School of Geosciences, University of Aberdeen, Meston Building, King’s College, Aberdeen AB24 3UE, UK; 3 Instituto Andaluz de Ciencias de la Tierra (CSIC-UGR), Armilla, 18100 Granada, Spain; 4 Centro de Astrobiología (CSIC-INTA), Torrejón de Ardoz, 28850 Madrid, Spain

**Keywords:** human vision, atmosphere, photopic vision, scotopic vision, evolution, astrobiology

## Abstract

Since the earliest development of the eye (and vision) around 530 million years ago (Mya), it has evolved, adapting to different habitats, species, and changing environmental conditions on Earth. We argue that a radiation environment determined by the atmosphere played a determining role in the evolution of vision, specifically on the human eye, which has three vision regimes (photopic-, scotopic-, and mesopic vision) for different illumination conditions. An analysis of the irradiance spectra, reaching the shallow ocean depths, revealed that the available radiation could have determined the bandwidth of the precursor to vision systems, including human vision. We used the radiative transfer model to test the existing hypotheses on human vision. We argue that, once on the surface, the human photopic (daytime) and scotopic (night-time) vision followed different evolutionary directions, maximum total energy, and optimum information, respectively. Our analysis also suggests that solar radiation reflected from the moon had little or no influence on the evolution of scotopic vision. Our results indicate that, apart from human vision, the vision of only a few birds, rodents, and deep-sea fish are strongly correlated to the available radiation within their respective habitats.

## 1. Introduction

Two photoreceptors (cone and rod cells) facilitate vision in most organisms with advanced visual system The vertebrate photoreceptors constitute four photopigments associated with cone cells and one photopigment associated with rod cells. The cone photopigments are expressed as short-wavelength sensitive-1 (SWS1), short-wavelength sensitive-2 (SWS2), rhodopsin-like, medium wavelength sensitive (RH2), and long wavelength sensitive (LWS). The rod photopigment is expressed as rhodopsin (RH1) [1]. The number, density, and anatomy of photoreceptors vary for different species, resulting in a contrasting vision regime. For example, most non-primate mammals have two cone photopigments, whereas human eyes have three. In addition, the spectral sensitivity of these photopigments varies between the species, depending on their anatomy and biochemical compositions [1,2,3,4]. Extensive research is available on the biological aspects, such as retinal physiology, anatomy, and underlying photodetection process in the eye [5,6,7,8].

Early eyes developed and evolved based on functional requirements and sensory tasks [9]. The onset of early vision, development, and evolution to high-resolution vision took approximately 170 million years during the Palaeozoic era before the onset of the Cambrian explosion (~530 Mya) [10]. Since the development of early eyes, Earth experienced five large extinction events: End-Ordovician (~443 Mya), Late-Devonian (~370 Mya), End-Permian (~252 Mya), End-Triassic (~201 Mya), and End-Cretaceous (~66 Mya) [11]. These extinction events caused drastic loss of life, potentially resetting the biological evolution and changes in the atmosphere over time. Thus, early vision and life itself did not seem to evolve gradually due to the abrupt changes invoked by the extinction events.

Early vision developed in oceans for the aquatic organisms, (from simple photodectection to complex image forming vision) [5,9,12], carried over to terrestrial life forms during their migration to land. The human eye further evolved on the surface, ever since humans came into existence after the End-Cretaceous extinction event. Therefore, to determine the influence of irradiation on the early evolution of vision systems—the radiation reaching the ocean’s depths and the surface radiation environment should be analysed. This protovision system should have impacted the ulterior evolution of sight once life evolved to the surface. Therefore, both environments must have left a footprint on the final characteristics of the current human vision. The spectral characteristics of the eye also depend on the radiation environment determined by the atmospheric conditions and the sun’s radiation.

The human eye could be considered a natural optical device with a very sophisticated data processor (the brain), which has co-evolved with humans. The spectral characteristics of human vision are in close resemblance to the maximum of the sun’s energy spectrum. The non-coincidental nature of human vision in the visible spectrum where the sun has its Wien’s peak is well established [13,14]. The human eye exhibits different vision regimes, namely photopic-, scotopic- and mesopic vision. During the well-illuminated daytime, photopic vision is activated by cone cells. The scotopic vision is activated in low-light conditions by rod cells [5,7,8,9]. The mesopic vision occurs in the intermediate illumination conditions where both the cone and rod photoreceptors are active [15].

During the photopic vision, the trichromatic vision of the human eye is facilitated by three photopigments with different wavelength sensitivities denoted as short (S), middle (M), and long (L) cone cells. These cone photopigments have peak absorptions at around 420, 530, and 559 nm, respectively, for S, M, and L cones [7,16]. The combined response from the three cone cells results in photopic vision with peak spectral sensitivity at 555 nm [17,18]. On the other hand, the rod cells facilitating scotopic vision only have one photopigment, rhodopsin, with peak absorption around 500 nm [14,19,20].

The wavelength range of human vision is generally accepted to be 400–700 nm with a spectral bandwidth of 300 nm [13,21]. However, some studies presented the vision range as between 380 and 760 nm, determined by bond dissociation energies [22]. On the other hand, few studies have presented the human eye’s sensitivity in the infrared region owing to nonlinear processes [21,23]. Therefore, we can conclude that strict wavelength limits of human vision, and the factors determining it, are not clearly defined yet.

Overduin (2003) noted that the peak absorption wavelength for photopic vision is determined by the maximum total energy of solar radiation for the 300 nm wavelength range of vision. To compensate for the atmospheric processes, such as Rayleigh scattering and the greenhouse effect, a 5500 K apparent effective temperature of the sun was considered [13]. Delgado-Bonal and Martín-Torres (2016) hypothesised that the peak absorption wavelength is determined by a compromise between the energy of photons reaching the eye and their information content, and proposed an optimum information wavelength calculated from combined energy and entropy distributions of radiation spectra at the surface. For this purpose, the surface radiance spectrum was calculated using a Planck function at an effective temperature of 5800 K, including the Rayleigh scattering function [24], and modelled the transmission spectra for present-day Earth atmosphere.

The maximum total energy hypothesis [13] and the optimum information wavelength hypothesis [14] are two different existing theories for determining the maximum absorption wavelengths of human vision regimes. Importantly, these two hypotheses do not consider the standard solar irradiance spectra (i.e., including the characteristic absorptions and emission peaks of the elements of the sun) and the complete array of atmospheric processes experienced by the radiation reaching the surface. Furthermore, the level of radiation reaching the first layers of water in oceans has not been considered, regarding its impact on the vision system.

In this article, we determine which of the two theories is applicable for different human vision regimes. We achieve validation of these theories using high-resolution modelled data representative of present-day Earth, while also overcoming the outlined shortcomings of the theories. This serves two purposes (1) it shows how closely the evolved human vision correlates to the present-day atmospheric conditions and (2) avoids uncertainties of the atmospheric conditions of the past eons. Additionally, the evolution process that has occurred continuously over millions of years, leading to present day vision characteristics, would be best validated with present-day conditions. We performed a detailed model of the irradiation levels, reaching the surface and the first layers of ocean depths, to understand the vision bandwidth and maximum absorption wavelengths of the human vision’s photopic- and scotopic-vision regimes.

## 2. Methods

We used the radiative transfer model, coupled ocean-atmosphere radiative transfer (COART), to calculate the required radiance spectra reaching the ocean’s surface and depths [25]. The COART model is publicly distributed from NASA, freely available for web-based online simulations (https://satcorps.larc.nasa.gov/jin/coart.html, last accessed: 2 December 2021). The model has multiple standard atmospheric profiles and parameters as inputs to calculate radiance reaching the surface and different depths of the ocean. In addition, the model uses the HITRAN 2000 molecular absorption database [26] for calculations, and restricts the fundamental radiative transfer code changes. The COART model was also chosen for its ease of reproducibility and availability.

All of the simulations were carried out for clear sky (cloudless, no aerosol) conditions. We also consider the direct downwelling irradiance and total downwelling irradiance (direct + diffuse). As per the requirements, some studies were conducted at the zero solar zenith angle (SZA) and some for high SZA (close to the horizon). The high-resolution (0.01 nm resolution) radiance spectra were calculated using COART, including the atmospheric processes, such as Rayleigh scattering and absorptions by all atmospheric species for standard mid-latitudes summer atmosphere [27]. We analysed the correlation of the modelled downwelling irradiance reaching the surface to the peak absorption wavelengths of human vision in photopic and scotopic vision regimes. The radiation available at different depths of the ocean was considered, to present its influence on the vision’s spectral bandwidth (range) in the visible spectrum. Spectral entropy spectra were calculated for the obtained radiance spectra to determine the optimum radiance distribution and optimum information wavelengths [14].

## 3. Results

The solar radiation passing through the Earth’s atmosphere undergoes reflection, absorption, and scattering on its way to reach the surface [28]. The radiance reaching the surface comprises two components, namely, direct-beam and diffuse radiation. Diffuse radiation is significant in overcast, cloudy conditions, whereas direct-beam radiance is predominant for the cloudless, clear sky conditions [29]. Clouds absorb and scatter solar irradiance, increasing the diffuse component of the downwelling radiation [28,30]. High volcanic activity during the late-Cambrian period resulted in mass extinctions of the death interval. After the reduced volcanic activity, the following great Ordovician biodiversification event (GOBE, ~470Ma) kick-started the evolution process [31]. The biodiversification events clearly correlate to the clear sky conditions. The cloud cover and overcast conditions are variable factors of the climate. Following the general norm in atmospheric and planetary studies, we performed all irradiance calculations for clear-sky conditions in this article. We considered the direct downwelling irradiance for photopic vision and total downwelling irradiance (direct + diffuse) for scotopic vision.

### 3.1. The Spectral Bandwidth of Human Vision (Δλ)

**Figure 1:**
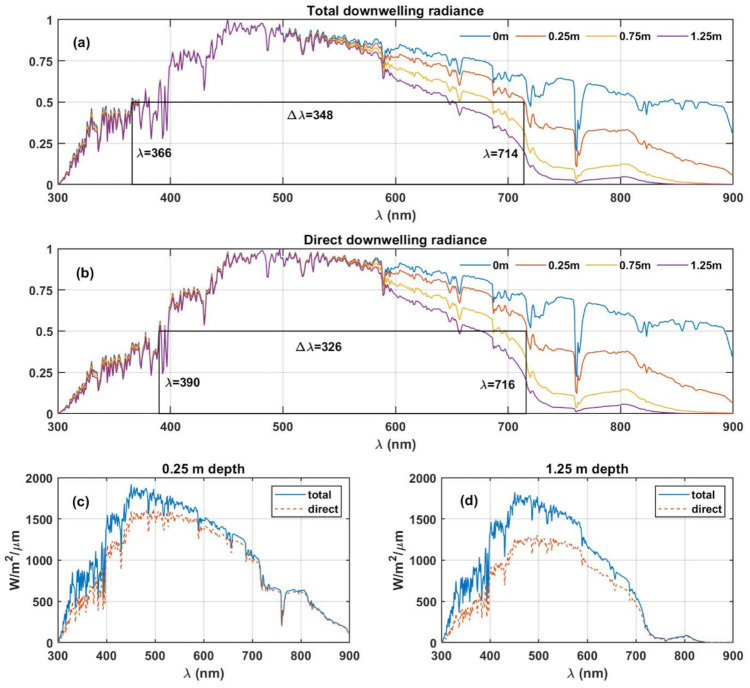
Solar radiation reaching the surface and down to the ocean at 1.25 m depth, including spectra at intermediate depths: 0.25 and 0.75 m. (**a**) Shows the total (direct + diffuse) downwelling radiance spectra at different ocean depths; (**b**) shows the direct downwelling radiance spectra at the corresponding levels. The rectangles indicated with wavelengths show the FWHM at 0.25 m ocean depth. (**c**,**d**) Show the differences between total and direct downwelling radiance at 0.25 and 1.25 m ocean depths.

We hypothesise that the extent/width of available radiance spectra could have a limiting effect on the wavelength range of the vision during its evolution. To investigate the possibility of the dependence of bandwidth of vision on the irradiance, we analysed the radiance spectra at Earth’s surface and down to the depth of 1.25 m (an arbitrary cut-off depth chosen for illustration) in the clear ocean (see Figure 1). The downwelling radiance presented in Figure 1 was obtained using COART at $SZA=0^{{}^{\circ}}$ in a standard mid-latitude summer atmosphere at 1 nm spectral resolution.

Figure 1a,b show the downwelling radiance spectra, starting from around 300 nm extending to 900 nm at 1.25 m depth and extending to longer wavelengths as the depth reduces. The O_2_ absorption band occurring at ~762 nm, clearly observable in surface radiance spectra, is attenuated by the absorption of the water column as the depth increases. The O_2_ absorption band undergoes complete attenuation at 1.25 m ocean depth (see Figure 1d) due to the absorption, in an increased length of the H_2_O column following the Beer–Lambert law. We calculate the full width at half maximum (FWHM) of the spectra. The FWHM is a widely used metric to represent bandwidth in communication and optical systems. We observe that this value decreases with depth within the aquatic environment. The numerical values of the FWHM at certain reference levels are summarised in Table 1. This observation shows that the eye is a natural photon detector that acts as a bandpass filter absorbing specific bandwidth of light to facilitate vision. Therefore, the FWHM of downwelling radiance could have determined its bandwidth, as shown in Figure 1b.

The results shown in Figure 1 and Table 1 indicate that depending on the depth, the available light environment in bandwidth moves to shorter wavelengths as the radiation penetrates the ocean, ranging from 369 nm at the surface to 265 nm at 1.25 m depth. Oceanic turbidity levels are hypothesised to have played a significant role in the diversification of animal species over time. During the maximum turbidity periods, the species with non-visual cues diversified, whereas, in the turbidity minima periods, species with visual systems developed and diversified [32]. Although, competing studies presented lack of correlation between oceanic turbidity/opacity and diversification of species with visual systems [33,34], the low oceanic turbidity would facilitate development of visual system as opposed to high turbidity conditions by allowing larger amounts of light to reach the depths of the ocean. Thus, low turbidity conditions are more conducive for the evolution of image-forming visual systems. Since the evolution of vision from protovision to full-scale image formation, human vision occurred over millions of years in changing ocean and atmospheric environments, and variable parameters, such as turbidity, cannot be accurately considered. Furthermore, due to varying oceanic turbidity over the evolution period, a comparison to the present-day oceanic turbidity levels could not be performed. Therefore, in this article, we considered pure, clear water to analyse the radiation reaching the shallow depths of the ocean. However, other products dissolved or in suspension in ocean water (such as minerals, soils, salts, etc.), should also affect the absorption bands, the scattering, and light transmission through the water column.

Furthermore, the life forms that developed early eyes during the evolution of vision (~170 million years) would certainly not have been confined at a constant depth in the ocean. Therefore, we cannot define a strict limit on the location (depth in the ocean) where early vision would have developed with our current knowledge. The calculations presented here describe the aquatic environment’s role on the final FWHM, qualitatively.

Following the assumptions described above, the generally accepted human vision bandwidth of 300 nm [13] would correspond to depths between 0.5 and 0.75 m in the ocean where the FWHM of available radiation correlates with the reported vision bandwidth. Thus, the bandwidth of available radiation (Table 1) for 300 nm of human vision suggests that the visual functions of the precursors of the human eye system may have been developed, to be optimal in shallow depths of aquatic environments, close to the surface.

Since the development of the primary visual system in the ocean, it is conceivable that the radiation environment available in the ocean’s depths could have determined the vision bandwidth. This would further increase the chances of migrating to land with well-adapted vision helpful in their survival and visually guided behaviour. The visual system of life forms that moved to the surface evolved for millions of years once the habitat moved to the surface. As brains evolved and signal analysis processes became sophisticated, it is reasonable to assume that other factors may have influenced the functionality of the full visual system, particularly those that included information. The following analysis focuses on how the visual system operates on the surface with the constraint of adhering to a fixed bandwidth.

### 3.2. Photopic Vision

In this section, we present our radiance calculations to compute the maximum absorption wavelength for photopic vision. We used COART to compute the high-resolution (0.01 nm) radiance spectra at the surface in clear-sky conditions for standard mid-latitudes summer atmosphere. The radiance spectra are obtained for $SZA=0^{{}^{\circ}},$ which represents the maximum possible radiation available. The use of high-resolution spectra facilitates the inclusion of representative atmospheric absorption features and better accuracy of the numerical integration of the radiation energy.

**Figure 2:**
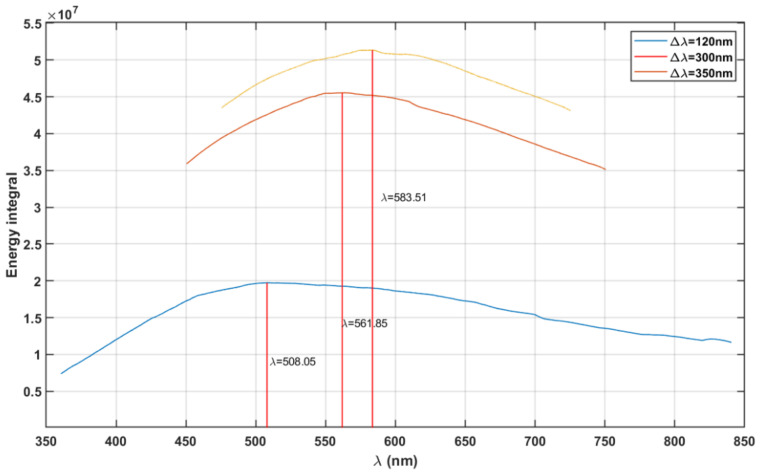
Intensity of radiation energy at each centre wavelength (λ) for 120, 300, and 350 nm vision bandwidth (Δλ).

Figure 2 shows the results for maximum absorption wavelength obtained from the maximum total energy-driven evolution hypothesis [13]. We perform the numerical integration of the irradiance spectra using the trapezoidal integration method. The energy integral at each centre wavelength (λ, varying from 300 to 900 nm) is calculated within limits ranging Δλ2 on either side, where Δλ is the considered bandwidth, i.e., 120, 300, and 350 nm. The vertical lines in Figure 2 represent the maximum total energy integral wavelength $\left(\lambda_{max}\right)$ at 561 and 583 nm for Δλ= 300 and 350 nm, respectively.

Assuming the human vision bandwidth range Δλ=300 nm, the evolution driven by maximum cumulative energy would have a photopic vision from 412–712 nm with maximum absorption $(\lambda_{max})\;$at 561.85 nm. Similarly, for bandwidth Δλ=350 nm, the photopic vision would be between 408 and 758 nm with maximum absorption $(\lambda_{max})\;$at 583.51 nm. Apart from the 300 nm bandwidth (i.e., of human vision), we selected two arbitrary bandwidth values, 120 and 350 nm, to represent the corresponding energy integrals showing the variance in maximum absorption wavelengths.

**Figure 3:**
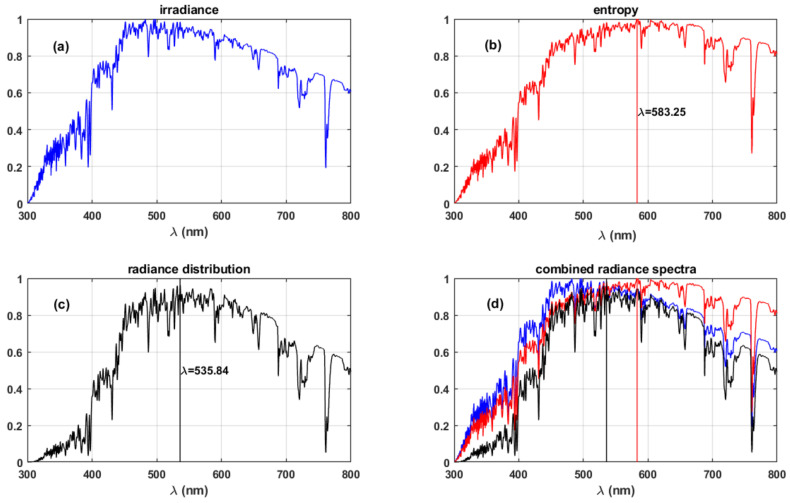
(**a**) Spectral energy, (**b**) spectral entropy, (**c**) radiance distribution, and (**d**) combined representation of the spectra for the radiation reaching the surface. The vertical lines in panels (**b**–**d**) represent the wavelengths of the maxima of the respective spectra.

Figure 3 shows the resulting spectra for the optimum information-driven evolution hypothesis [14]. Figure 3a shows the radiance spectra at the surface. Spectral entropy (S) spectra obtained using Equation (1):

(1) $$S_{\lambda }=\frac{2kc}{\lambda^{4}}\left\{\left(1+\frac{\lambda^{5}L_{\lambda }}{2hc^{2}}\right)\ln\left(1+\frac{\lambda^{5}L_{\lambda }}{2hc^{2}}\right)-\left(\frac{\lambda^{5}L_{\lambda }}{2hc^{2}}\right)\ln\left(\frac{\lambda^{5}L_{\lambda }}{2hc^{2}}\right)\right\}$$

where k is the Boltzmann constant, c is the speed of light, h is the Planck’s constant, $L_{\lambda }$ represents radiance at wavelength λ, is shown in Figure 3b.

Figure 3c shows the combined radiance distribution obtained from the relation shown in Equation (2):

(2) $$\frac{S}{S_{max}}*\frac{L}{L_{max}}$$

where, $S_{max}$ and $L_{max}$ represent the maximum of the entropy and energy spectra. *L* is the radiance spectra obtained from COART simulations shown in Figure 3a. Figure 3d presents the combined representation of the radiance, entropy, and radiance distribution spectra.

The relation shown in Equation (2) represents the radiance distribution, which is the product of the normalised radiance and entropy spectra. The optimum information wavelength is defined as the wavelength at which the radiance distribution is maximised [14]. Our results in Figure 3 indicate that the optimum wavelength for human vision, with a bandwidth of 300 nm, occurs at 535.84 nm during photopic vision.

The previous studies and our analysis in this article are based on present-day earth conditions. The new results obtained in our analysis using a more representative radiation environment differ from the initial results presented by the respective authors due to the constraints of their assumptions. The difference in the obtained maximum absorption wavelengths for photopic vision is summarised in Table 2. Delgado-Bonal and Martín-Torres (2016) considered a black body radiation spectrum from Planck’s function and the corresponding spectral entropy spectra rather than the standard solar irradiance spectra; this is the primary factor for the difference in the results. Lack of consideration for atmospheric absorption features in the surface radiance spectra and standard solar irradiance spectra by Overduin (2003) induce small offsets in the final results compared to our analysis. The absence of strong absorption features by atmospheric species in the visible spectrum and good numerical estimation of the greenhouse effect in the atmosphere makes the maximum total energy hypothesis consistent with the observed human photopic vision parameters.

The new results we present in this work, with the improved atmospheric radiation data, show that the optimum information hypothesis would result in maximum absorption wavelength at ~536 nm, which is 20 nm short of the actual maximum absorption wavelength during human photopic vision (listed in Table 2). For human-like lifeforms on planets around sun-like stars (G-type), this difference would not have significant impact, since the visible band of EM spectrum does not have significant absorption features (Figure 3). The same will have severe implications for planetary systems around M-type stars, which have downwelling radiation predominantly in the infrared region, where most atmospheric gases show strong absorption features.

Furthermore, this difference of ~20 nm becomes significant when the combined response of the three cone cells: S, M, and L are taken into consideration. These cone cells of the human eye have maximum sensitivities around 420, 530, and 559 nm, respectively [16]. The discrepancy in the result affects the contributions of the individual cone cells to the overall spectral response of the eye. The maximum absorption wavelengths, 536 nm (optimum information) and 561 nm (maximum total energy) indicate M cone cells and L cone cells, respectively, would have significant contribution to the overall spectral response. The observed maximum absorption wavelength for the photopic vision at 555 nm [18] would mean L cone cells dominate the individual contribution from the three types of cone cells.

Therefore, from the analysis of modelled data representing practical, atmospheric conditions, we conclude that the evolution of photopic vision was directed towards maximising the total energy within the bandwidth of the vision.

### 3.3. Scotopic Vision

The rod cells in the human eye that facilitate scotopic vision are highly sensitive to light and are almost 20 times more than cone cells. They also have different anatomy to cone cells [5,35,36]. Given that the photoreceptors responsible for the human photopic and scotopic vision are physiologically different, the two vision regimes could have evolved in different paths to maximise the use of human vision under different environmental conditions. The optimal functionality of rod cells occurs close to dusk and dawn.

**Figure 4:**
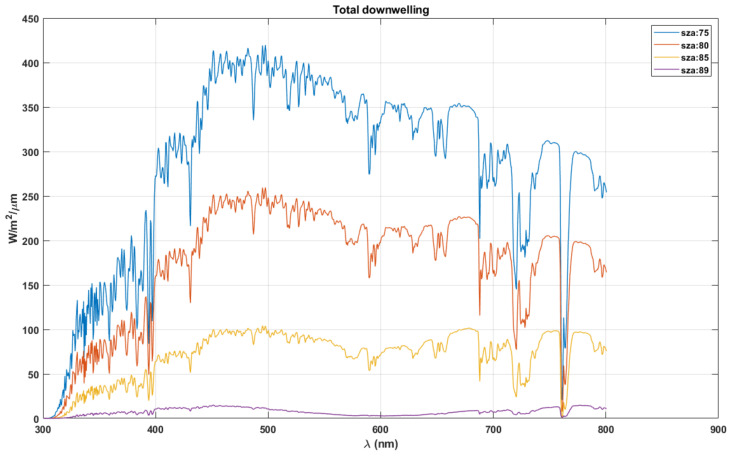
Total downwelling solar radiation reaching the surface of the Earth during dusk/dawn time of the day.

For the first time, we present the possible correlation of human scotopic vision to the irradiance levels at different SZAs. The maximum and minimum solar radiation at the surface of the Earth occur at 0° and 90° SZA, respectively. As the SZA reaches its maximum and the sun is close to the horizon, the diffuse radiation component becomes significant, decreasing the direct radiation component. Therefore, we consider for our analysis the total downwelling radiance at high SZA for scotopic vision. Figure 4 shows the total downwelling radiation at late dusk and early dawn when the mesopic vision is prevalent, with both cone and rod cells being active. The spectra are obtained using COART for a clear-sky, no aerosol standard mid-latitude summer atmosphere (45° N).

The existing hypothesis states that the optimum information wavelength determines the human scotopic vision [14] to optimise information content for the vision. Therefore, we consider the total downwelling radiation at dusk/dawn to establish the hypothesis on the evolution of scotopic vision. Figure 4 shows that, as the SZA increases from 75° to 89°, after 80°, the radiation at the longer wavelengths (>600 nm) become stronger than at the short wavelengths. Therefore, we choose the downwelling radiation at 80° SZA for analysis to avoid skewing results, considering the peak absorption during scotopic vision occurring around 500 nm.

The human eye’s rod spectral sensitivity function indicates that the scotopic vision occurs approximately between 400 and 600 nm with 200 nm bandwidth [37]. However, our analysis of the radiance spectra with the maximum total energy-driven evolution hypothesis [13] resulted in a maximum absorption wavelength at 542 nm for 200 nm vision bandwidth (see Supplementary Materials). Therefore, the maximum total energy hypothesis does not fit the evolution of scotopic vision as it does to the photopic vision.

**Figure 5:**
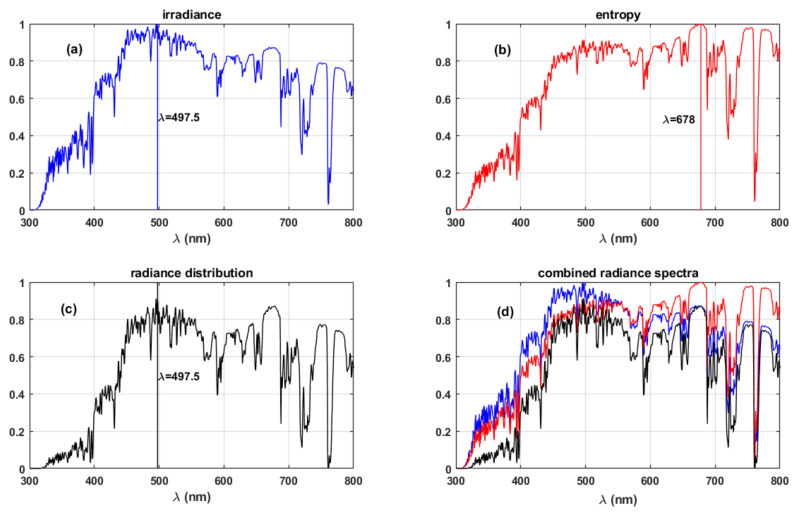
Total downwelling radiation at SZA: 80°. (**a**) Spectral energy, (**b**) spectral entropy, (**c**) radiance distribution and (**d**) combined representation of the spectra for the radiation reaching the surface. The vertical lines in panels (**a**–**c**) represent the wavelengths of the maxima of the respective spectra.

Figure 5 shows the downwelling radiation at 80° SZA and corresponding spectral entropy and radiance distribution spectra obtained from Equations (1) and (2), respectively. The results show that both the maximum irradiance and radiance distribution corresponding to optimum information have the maximum at 497.5 nm, emphasising the superior contribution of spectral radiance than spectral entropy for this radiation environment. Furthermore, the possible maximum absorption wavelength during human scotopic vision at 497.5 nm (Figure 5) correlates to rod cells’ maximum sensitivity wavelength, 496 nm, as reported in [20,38].

Observing the spectra in Figure 5, both the maximum energy-driven (not maximum total energy) and optimum information-driven evolution seem plausible for human scotopic vision. However, while either evolutionary direction has equal possibilities, the information-optimised evolution would be particularly efficient, as it encompasses the maximum energy criterion in addition.

**Figure 6:**
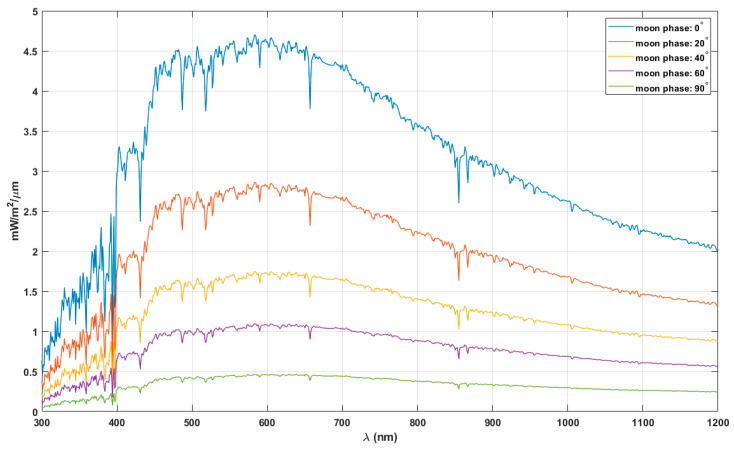
Lunar spectral irradiance at different phases of the moon at standard geometry conditions and mean Moon-Earth distance.

It is prudent to investigate the possible influence of light from the moon, which constitutes a significant aspect during the night wherein the scotopic vision is prevalent. Here we consider the lunar irradiance to investigate its impact on the scotopic vision when the sun is below the horizon. The lunar spectral albedo is wavelength dependent, and it increases with the wavelength in UV and visible bands [39]. This results in the lunar irradiance spectrum being different to that of solar irradiance. The solar radiation reflected by the moon and reaching the surface of the Earth varies significantly in intensity with respect to the phase of the moon and the geometric alignment of the three bodies. Figure 6 shows the lunar spectral irradiance at TOA during different phases at standard geometry conditions and mean Moon–Earth distance [39].

Figure 6 shows that the mean lunar spectral irradiance peaks at wavelengths ~600 nm, far from the maximum sensitivity wavelength during scotopic vision. The results from the analysis of the lunar irradiance spectra with both optimum information hypothesis and maximum total energy hypothesis showed that the maximum absorption wavelengths are well above 600 nm (see Supplementary Material). These results indicate that the solar radiation reflected from the moon, reaching the surface of the Earth, had little or no influence on the evolution of scotopic vision. The daily variation of the lunar irradiance w.r.t to phase in contrast to the relatively stable solar radiation during the latter part of the day could have led to the evolution of human scotopic vision with little dependence on the irradiation from the moon.

## 4. Discussion

### 4.1. Vision of Other Species

The visual systems are species-specific, and the evolution of vision is determined by the anatomy, biochemical composition, habitat, behavioural patterns, and the available radiation environment. In general, nocturnal species have high rod cell density and lower cone cell numbers than diurnal species. Some species, such as bees, birds, some rodents, and few bat species have UV-sensitive photopigments [1,2,3,40,41]. The UV sensitivity of terrestrial nocturnal species could result from the evolution from the early vision in the oceans where they had exposure to UV radiation.

Here, we discuss few animal species, showing features of their visual system closely correlated with the radiation environment. This shows that other animal species apart from humans have also developed their image-forming vision in correlation with the radiation environment. Dedicated species-specific studies would shed more light on the evolution of their vision to atmospheric conditions. The spectral sensitivities of M cones of three rodent species, gerbils, Wistar rats, and mice, have peaks at 511, 493, and 502 nm, respectively [42]. From Figure 2, we find that the peak spectral sensitivities of gerbils and mice closely match with the vision for Δλ=120 nm (~440–560 nm) with maximum absorption at 508 nm. Whereas the peak spectral sensitivity of Wistar rats matches with the absolute maximum of the direct radiance spectrum at 497.5 nm. Considering similar evolution of the visual system for the rodent species, the M cone photopigments of these rodent species could have evolved to maximise the total energy like the photopic vision of humans.

**Figure 7:**
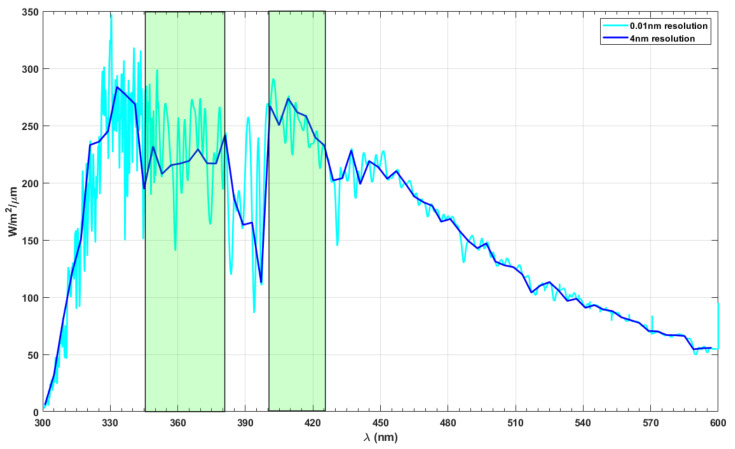
Diffuse radiance reaching the surface of the Earth. A high resolution (0.01 nm) and low-resolution (4 nm) spectra are shown in the graph to highlight distinct regions at 350–380 nm and 400–420 nm.

The UV-sensitive photopigments of birds have maximum sensitivities around 360–373 nm and 402–426 nm [41]. These bands appear to fall within the distinct regions with stable irradiance seen in the diffuse radiance spectrum shown in Figure 7. The low-resolution (4 nm) spectrum of diffuse radiance is utilised to identify and highlight the two regions with relatively stable energy. Deep-sea pearlside fish that migrate close to the surface during dusk and dawn have vision with peak spectral sensitivity at 494 nm, similar to the scotopic vision of the human eye [20]. The visions of pearlside and scotopic vision of humans are active during the intermediate illumination conditions and closely correlate to the radiation environment following optimum information-driven evolution (Figure 6).

### 4.2. Sources of Uncertainties

Protovision developed ~530 Mya and evolved to the current human visual system, adapting to the changing environmental conditions and habitat. The changes in the atmosphere and radiation environment throughout millions of years would undoubtedly influence the eye’s evolution. The changes in the terrestrial landmass over the years (from the Pangea to the current continental map) resulted in different radiation environments available for the species. By the End-Cretaceous period, the landmass distribution put the continental plate of Africa (origin of homo sapiens) near the equator [32,43]. The human eye development would have been completed in this region before migrating to other continental plates.

The evolution and its response to the changes in the environment are slow processes. Therefore, the primary sources of uncertainties in studying the evolution of a visual system are the different environmental conditions driven by changes in the atmosphere and solar radiation in the past.

The sun’s evolution over different geological periods resulted in changes in spectral distribution and the magnitude of solar radiation. The young sun was 25% less luminous and had ten times stronger UV flux [44]. The modelled solar UV irradiance spectra for 3.9 Ga and 2.0 Ga show stark variations, whereas the same for 0.8 Ga and the modern-day sun are closely matched [45,46]. This shows that the sun’s evolution does not contribute to the uncertainties in human vision (as it evolved much later). While analysing irradiance spectra, the spectral resolution could result in large offsets in the results. A high-resolution spectrum comprises absorption features of the atmospheric constituents. In contrast, a low-resolution spectrum suppresses the spectral features in the surface radiance spectra, overruling the impact of atmospheric absorbers.

## 5. Conclusions

The impact of the atmosphere on the evolution of human vision is a sparsely studied area compared to the biological aspects. In this article, we investigated the limiting effect of radiation reaching ocean depths, determining the bandwidth of human vision. The FWHM of the irradiance at the ocean depths corresponds to the human eye’s observed spectral bandwidth, suggesting that its evolution started in shallow depths of the ocean. Furthermore, validating the existing hypotheses on the evolution of human vision with the representative radiance spectra indicates that the photopic and scotopic vision regimes may have resulted from different evolutionary directions. The results indicate that the human photopic vision evolved towards maximising the total energy within the limits of the vision bandwidth. On the other hand, the human scotopic vision evolved towards optimising information content with respect to solar irradiance (and not lunar irradiance).

The visual systems of other species, such as rodents, birds, and deep-sea fish also strongly correlate to the atmospheric radiation environments. Our results show that the radiation environment defined by atmospheric compositions is detrimental to the evolution of visual systems. The close correlation of spectral characteristics of human vision to the radiation environment indicates that atmospheric conditions and biological development go hand-in-hand during the evolution process.

## Figures and Tables

**Table 1:** FWHM wavelengths of the radiance spectra at the surface and different ocean depths for the total downwelling and direct dowelling radiance.

Depth (m)	Total Downwelling Radiance		Direct Downwelling Radiance	
	Wavelengths (nm)	FWHM (nm)	Wavelengths (nm)	FWHM (nm)
1.25	366–647	281	390–655	265
1.00	366–655	289	390–686	296
0.75	366–686	320	390–686	296
0.50	366–686	320	390–709	319
0.25	366–714	348	390–716	326
0.10	366–716	350	390–718	328
surface	366–718	352	390–759	369

**Table 2:** Results for the maximum absorption wavelengths from the two hypotheses and their respective results obtained in this work with the high resolution, representative atmospheric radiation data as outlined in the methods section.

**Maximum Total Energy Hypothesis** Δλ=300 nm		**Optimum Information Hypothesis**		**Photopic Vision**
Overduin, 2003	This work	Delgado-Bonal and Martin-Torres, 2016	This work	
560 nm	561.85 nm	555 nm	535.84 nm	555 nm

## Data Availability

Not applicable.

## Supplementary Materials

The following are available online at https://www.mdpi.com/article/10.3390/life11121337/s1, Figure S1: Scotopic vision-Energy integral distribution for the considered vision bandwidths, Figure S2: Energy integral distributions of lunar irradiance, Figure S3: Irradiance, Spectral entropy, and optimum radiance distributions of Lunar irradiance along with the wavelengths of corresponding maxima.
