# Supplementary material for: The Impact of the Spectral Radiation Environment on the Maximum Absorption Wavelengths of Human Vision and Other Species
Source: Life (Basel). 2021 Dec 3;11(12):1337. doi: 10.3390/life11121337 (PMC8707699; doi:10.3390/life11121337)
Supplement: Supplementary file 1 [file life-11-01337-s001.zip › life-1480479-supplementary.pdf]

Supplementary Materials

The Impact of the Spectral Radiation Environment on the Maximum Absorption Wavelengths of Human Vision and Other Species

Scotopic vision

Figure S1 shows the results from the maximum total energy hypothesis for scotopic vision. The results indicate that this hypothesis does not confer to the scotopic vision of human eye.

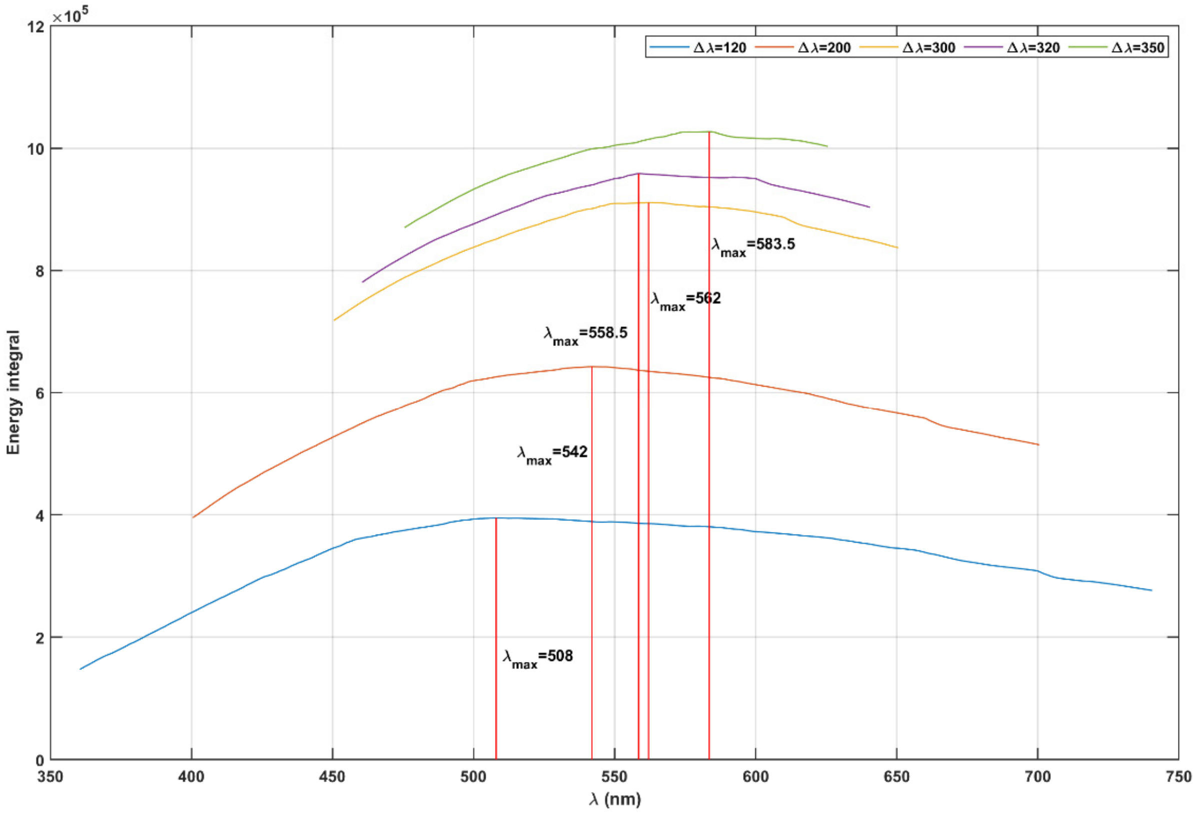

Figure S1. Scotopic vision - Energy integral distribution for the considered vision bandwidths.

Figure S2 shows the energy integral distributions of the lunar irradiance for different vison bandwidths considered.

Figure S3 shows the lunar irradiance and corresponding spectral energy and optimum radiance distributions following the optimum information driven hypothesis.

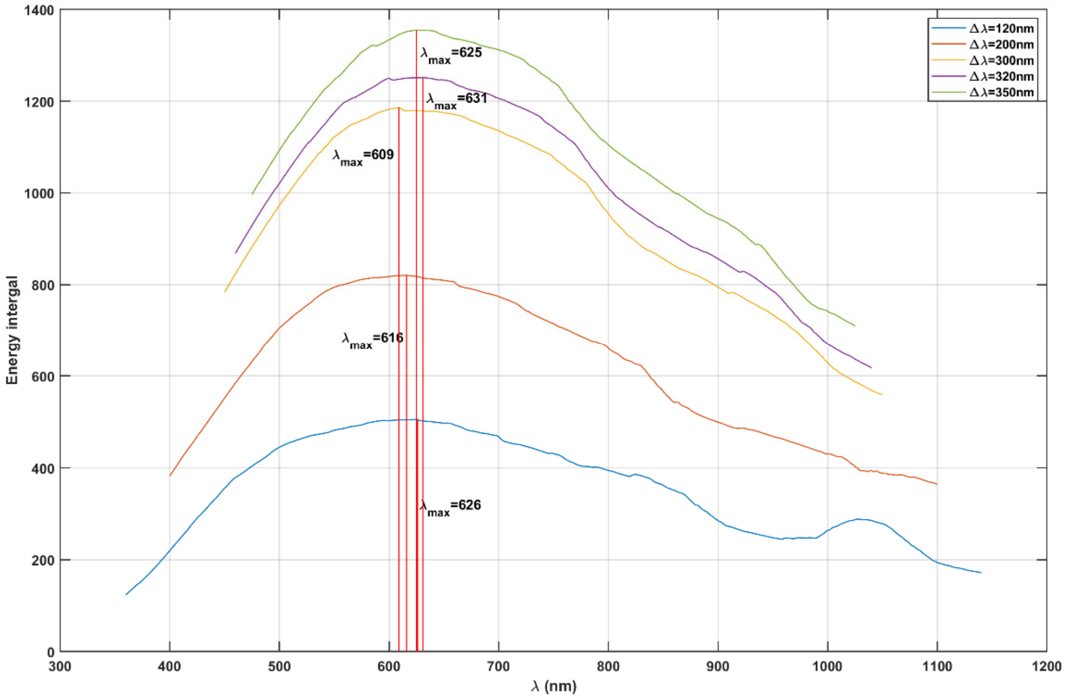

Figure S2. Energy integral distributions of lunar irradiance. The vertical lines denote wavelengths of maximum total energy integrals.

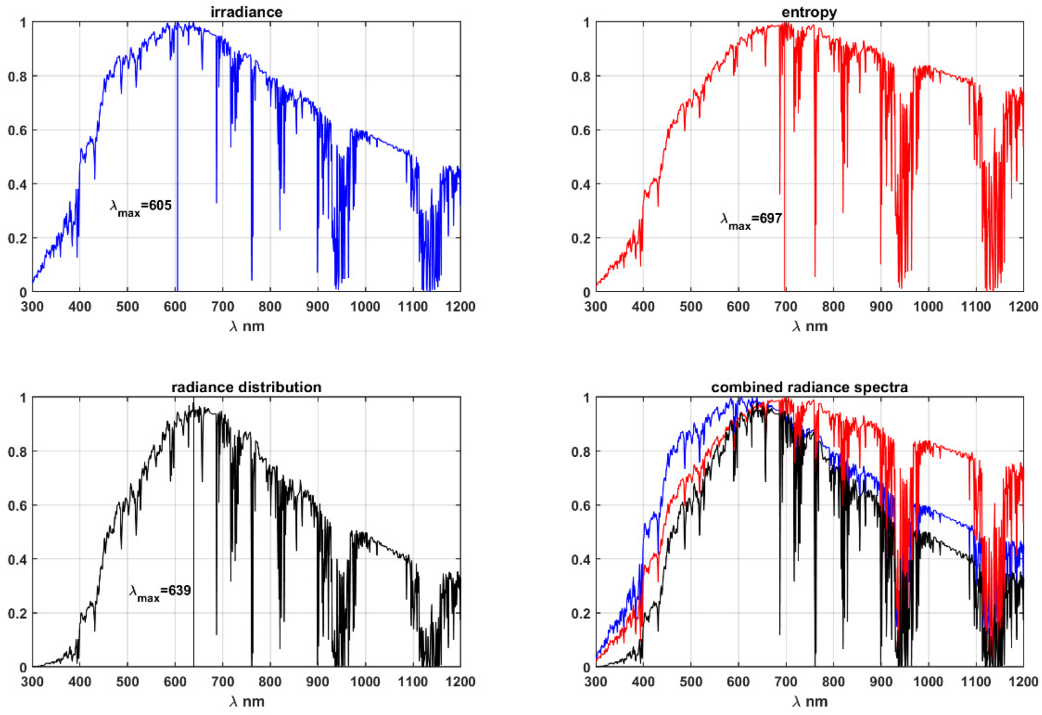

Figure S3. Irradiance, Spectral entropy, and optimum radiance distributions of Lunar irradiance along with the wavelengths of corresponding maxima.
